# Effectiveness and safety of botulinum toxin injection in the treatment of recurrent anal fissure following lateral internal sphincterotomy: cohort study

**DOI:** 10.1093/bjsopen/zrad156

**Published:** 2024-02-07

**Authors:** Giuseppe Brisinda, Valeria Fico, Giuseppe Tropeano, Gaia Altieri, Maria Michela Chiarello

**Affiliations:** University Department of Translational Medicine and Surgery, Catholic School of Medicine, Rome, Italy; Emergency and Trauma Surgery Unit, Department of Abdominal and Endocrine Metabolic Medical and Surgical Sciences, Fondazione Policlinico Universitario A Gemelli, IRCS, Rome, Italy; Emergency and Trauma Surgery Unit, Department of Abdominal and Endocrine Metabolic Medical and Surgical Sciences, Fondazione Policlinico Universitario A Gemelli, IRCS, Rome, Italy; Emergency and Trauma Surgery Unit, Department of Abdominal and Endocrine Metabolic Medical and Surgical Sciences, Fondazione Policlinico Universitario A Gemelli, IRCS, Rome, Italy; Emergency and Trauma Surgery Unit, Department of Abdominal and Endocrine Metabolic Medical and Surgical Sciences, Fondazione Policlinico Universitario A Gemelli, IRCS, Rome, Italy; General Surgery Operative Unit, Department of Surgery, Provincial Health Authority, Cosenza, Italy

## Introduction

Lateral internal sphincterotomy is an effective treatment for anal fissure^1–3^. Despite the high success rate of anal fissure healing after lateral sphincterotomy, recurrence may occur between 1.6 and 6%^4,5^. Anal fissures that fail to heal or that recur after lateral sphincterotomy have been associated with an inadequate sphincterotomy or chronic morphological changes of the internal anal sphincter within the fissure, including fibrosis^6,7^.

Up to 47.6% of patients develop postoperative disturbance of continence after lateral internal sphincterotomy^8,9^. Long-term risk of incontinence is reported at an average of 14%^4,10,11^. In these patients, the sphincterotomy was found to be excessive or associated with a defect of the external sphincter^12^.

The poorly defined risk following incontinence of surgical sphincterotomy has increased interest in pharmacologic approaches to produce reversible reduction of sphincter pressure and obtain fissure healing minimizing incontinence risk^12,13^. Botulinum toxin has achieved the lowest rates of recurrence with the fewest side effects^14^. In a previous study, the authors evaluated the efficacy of botulinum toxin in the treatment of recurrent anal fissure^15^. The current study was designed to assess the efficacy of a higher dose of toxin in healing recurrent anal fissure following lateral internal sphincterotomy.

## Methods

Over a 10-year interval (2010–2019), patients with anal fissure who had previously undergone lateral internal sphincterotomy were recruited for the study at the Fondazione Policlinico Universitario Agostino Gemelli Istituto di Ricerca e Cura a Carattere Scientifico (IRCCS). All the patients underwent a pretreatment evaluation, including clinical inspection and anorectal manometry. Anorectal manometry was performed at rest and after voluntary contraction and was compared with the normal range for the authors’ laboratory. Resting anal tone and maximal squeeze pressure (the maximal voluntary increase above the resting tone) were determined by a station pull-through technique with three catheters water-perfused at a rate of 0.5 ml/min by a pneumo-hydraulic pump. A single operator performed all manometries. Each of the participants, lying on their left side, received a total of 100 U Dysport® injected with a 27-G needle in the internal anal sphincter, in two sites, on either side of the anterior or posterior midline depending on the location of the anal fissure (anterior injections for posterior fissure and posterior injections in the case of anterior fissure). No sedation or local anaesthesia was used during the procedure. No patient was treated with topical anaesthetic agents before or during the study. If the fissure persisted at the 2-month evaluation, the examiners could decide to re-treat a patient (rescue treatment). The re-treated patients received 150 U Dysport®.

Study end-point was the evaluation of complete healing at 1 month and 2 months after botulinum toxin injections. The results were expressed as mean (s.d.); differences between mean (s.d.) data were compared by the Student’s *t* test, while differences between percentages were analysed using the Fisher’s exact test. *P* < 0.05 was considered statistically significant. The research protocol was submitted to the local IRB. Ethical review and approval were waived for this study as it is a retrospective analysis of deidentified data.

## Results

Seventy-seven patients were assessed for eligibility: of them, six patients refused to participate. All 71 patients included in the study reported a history of previous lateral internal sphincterotomy for chronic anal fissure (performed 37.0(12.5) months before inclusion in the study) and complained of severe postdefaecatory pain. The baseline characteristics are reported in *Table 1*. The injections of the botulinum toxin were easily performed in all the patients. There were no complications observed in any of the patients during the procedure or postinjection side-effects.

**Table 1:** Characteristics at baseline of patients with relapse of chronic anal fissure following lateral internal sphincterotomy treated with botulinum toxin

Characteristics	Values
**Sex**	
Male	30
Female	41
Age (years), mean (s.d.)	43.3 ( 17.2)
Duration of symptoms, mean (s.d.)	7.4 (2.3)
**Anal pain**	
Postdefaecatory pain	71 (100)
Nocturnal anal pain	14 (19.7)
Unrelated to defaecation	3 (4.2)
Bleeding	51 (71.8)
**Defaecation pattern**	
Constipation	48 (67.6)
Diarrhoea	4 (5.6)
Lumpy or hard stool	68 (95.7)
Straining	59 (83.1)
Sensation of incomplete evacuation	24 (33.8)
Laxative use	35 (49.3)
Enema use	17 (23.9)
**Clinical examination**	
Surgical scar	60 (84.5)
Posterior anal fissure	57 (80.3)
Anterior anal fissure	14 (19.7)

Values are *n* (%) unless otherwise stated.

Clinical outcomes and anal pressures at 1-month and 2-month follow-up visits are shown in *Table 2*. One month after the treatment, inspection revealed a healing scar in 63 patients (88.7%). Five patients (7.0%) had mild flatus incontinence that lasted a few weeks and disappeared spontaneously. At the 2-month evaluation, 67 patients (94.3%) had healing scars and no patient complained of flatus incontinence. At the 1-month follow-up, resting anal pressure was lower than baseline values (*P* = 0.0001). At the 2-month evaluation, resting anal pressure was lower than the pretreatment value (*P* = 0.0001), while maximum voluntary squeeze pressure did not differ from baseline values.

**Table 2:** Clinical outcomes and anal pressures before and after botulinum toxin treatment in patients with recurrent anal fissure following lateral internal sphincterotomy

Clinical and manometric features	Baseline values	1-month values	2-month values
Anal fissure	71	8	4
Postdefaecatory pain	71	6	4
Nocturnal anal pain	14	0	0
Anal pain unrelated to defaecation	3	1	0
Bleeding	51	6	4
Resting anal pressure (mmHg), mean (s.d.)	97 (21)	75 (20)*	77 (19)*
Maximum voluntary squeeze pressure (mmHg), mean (s.d.)	82 (27)	79 (24)	81 (17)

Values are number of patients unless otherwise stated. All patients were included in all evaluations. *P = 0.0001 for the comparison with the baseline values (by Student’s *t* test).

A rescue treatment with 150 U of botulinum toxin was performed to the 4 patients who still had anal fissure 2 months after the first injection. In these patients, postdefaecatory pain was present in four, nocturnal pain in two and anal bleeding in four. One month after the rescue treatment, fissure healing was observed in all patients. At the same time, two patients complained of flatus incontinence that lasted a few months and spontaneously disappeared. At the 1-month evaluation, resting anal pressure and maximum voluntary squeeze pressure decreased from 93(14) mmHg to 79(14) mmHg and from 84(20) mmHg to 58(19) mmHg respectively. At the 2-month evaluation maximum voluntary pressure did not differ from the baseline value (85(18) mmHg), and resting anal pressure was 69(19) mmHg (*P* = 0.08 *versus* baseline values).

Clinical control was carried out by telephone interviews (once a year). All patients were given a dedicated telephone number to refer to in the event of specific symptoms. Clinical visit was performed only in patients who complained of specific symptoms. Complete information for the first 2 years after treatment was obtained from 68 patients. None of them reported the appearance of symptoms related to anal fissure and/or faecal incontinence, defined as uncontrolled losses of gas, mucus and/or liquid stools.

## Discussion

The aim of the treatment for chronic anal fissure is to reduce the tone of the internal anal sphincter^2,3,16,17^. Lateral internal sphincterotomy has been considered the standard approach. However, concerns have been raised about postoperative impairment of faecal incontinence after surgical sphincterotomy and about persistence or recurrence of the anal fissure. The risk of permanent incontinence after lateral sphincterotomy has stimulated interest in chemical sphincterotomy. In the current study 71 patients, who had previously undergone lateral internal sphincterotomy and presented with relapse of anal fissure, were treated with injection of botulinum toxin. At the 2-month evaluation, 67 patients had healing scars and five patients complained of incontinence to flatus which spontaneously resolved.

The healing rate (94.3%) was higher compared with previous experience^15^. This finding is likely related to the higher toxin dosage and different injection sites used in the current study. Choosing different injection sites could improve the clinical outcomes and induce a greater decrease in resting anal pressure^18,19^. However, despite having used a higher dose of toxin, there was not a similar decrease in resting anal pressure. It is likely that fibrosis induced by surgical sphincterotomy associated with fibrosis of the internal anal sphincter in the area of the fissure altered the diffusion of botulinum toxin. Choosing an injection site different from the site of the fissure was based on the concept that fibrosis of the internal sphincter is more prominent in the zone of the anal fissure than in other sites in the smooth muscle^20^. The fibrosis may reduce compliance of the internal sphincter and block the action of the toxin^14,19,21,22^. In patients with posterior chronic anal fissures, anterior injection was more effective in inducing healing than posterior injection^18^.

The increase of incontinence to flatus in patients with relapse of fissure after lateral internal sphincterotomy treated with botulinum toxin may be caused by damage induced by the surgical sphincterotomy of the whole internal sphincteric structural pattern, including the intrinsic innervation. Manometric experiences showed not only a rapid pressure decline after lateral sphincterotomy but several manometric fluctuations that lasted 1 year^23^. In this scenario, botulinum toxin injected into the internal anal sphincter in sites far away from the fissure is effective in treating patients with recurrent anal fissures and avoids permanent complications. Furthermore, the injection of the toxin was not only a therapeutic tool but also a diagnostic test to identify patients who would not be suitable for further sphincterotomy.

## Data Availability

The authors confirm that the data supporting the findings of this study are available within the article.

## Declarations

All procedures followed were in accordance with the ethical standards of the responsible committee on human experimentation (institutional and national) and with the Helsinki Declaration of 1964 and later versions. This article does not contain any experimental studies with human participants or animals performed by any of the authors.
